# 3D bioprinting technology to construct bone reconstruction research model and its feasibility evaluation

**DOI:** 10.3389/fbioe.2024.1328078

**Published:** 2024-01-19

**Authors:** Xiao Lv, Chenyang Zhang, Xingzhu Liu, Ping Li, Yadong Yang

**Affiliations:** ^1^ School of Laboratory Medicine and Bioengineering, Hangzhou Medical College, Hangzhou, China; ^2^ West China Hospital, Sichuan University, Hangzhou, China

**Keywords:** 3D bioprinting, bone reconstruction, cell bio-scaffolds, hydroxyapatite, sodium alginate, gelatin, polycarbonate membrane

## Abstract

**Objective:** To explore and construct a 3D bone remodeling research model displaying stability, repeatability, and precise simulation of the physiological and biochemical environment *in vivo*.

**Methods:** In this study, 3D bioprinting was used to construct a bone reconstruction model. Sodium alginate (SA), hydroxyapatite (HA) and gelatin (Gel) were mixed into hydrogel as scaffold material. The osteoblast precursor cells MC3T3-E1 and osteoclast precursor cells RAW264.7 were used as seed cells, which may or may not be separated by polycarbonate membrane. The cytokines osteoprotegerin (OPG) and receptor activator of NF-κB ligand (RANKL) were used to induce cell differentiation. The function of scaffolds in the process of bone remodeling was analyzed by detecting the related markers of osteoblasts (alkaline phosphatase, ALP) and osteoclasts (tartrate resistant acid phosphatase, TRAP).

**Results:** The scaffold showed good biocompatibility and low toxicity. The surface morphology aided cell adhesion and growth. The scaffold had optimum degradability, water absorption capacity and porosity, which are in line with the conditions of biological experiments. The effect of induced differentiation of cells was the best when cultured alone. After direct contact between the two types of cells at 2D or 3D level, the induced differentiation of cells was inhibited to varying degrees, although they still showed osteogenesis and osteoclast. After the cells were induced by indirect contact culture, the effect of induced differentiation improved when compared with direct contact culture, although it was still not as good as that of single culture. On the whole, the effect of inducing differentiation at 3D level was the same as that at 2D level, and its relative gene expression and enzyme activity were higher than that in the control group. Hence the scaffold used in this study could induce osteogenesis as well as osteoclast, thereby rendering it more effective in inducing new bone formation.

**Conclusion:** This method can be used to construct the model of 3D bone remodeling mechanism.

## 1 Introduction

Bone defect is a common and significant clinical problem. It may be caused by infection, tumor, trauma and congenital diseases which destroy the integrity of bone structure. At present, the main method for the treatment of bone defects is bone transplantation, which may be autogenic, allogeneic or artificial (Wang et al., 2020). Autogenous bone graft has definite curative effect and good compatibility. While the source of allogeneic bone may be varied, and the mechanical strength of artificial bone is high, these methods have some disadvantages, including long treatment cycle, complex technical requirements, sacrifice of healthy tissue, limited source of donors, immune rejection, and an inability to degrade to form new bone tissue (Lee et al., 2019; Mano et al., 2020; Zhu et al., 2020). The above mentioned transplantation methods have their own advantages and disadvantages. There is a lack of reliable data repair and reconstruction processes of bone after transplantation. The main reason for this could be the absence of a suitable and economically viable research model. At present, the research on the mechanism of bone resorption is mainly performed using 2D cell culture models or in animals *in vivo*. The 2D plane model detection method cannot adequately reproduce the 3D space environment, causing the cells to lose their inherent characteristics and functions (Hirschhaeuser et al., 2010). The 2D and 3D tissue models have different cellular responses, as demonstrated by the differences in their protein and gene expression, protein gradient profiles, cell signals, migration and invasion, morphology, proliferation, viability, tissue and drug responses (Wang et al., 2014; Ozbolat et al., 2016; Booij et al., 2019). Hence, the 2D cell models fall short of fulfilling the needs of advanced research, which subsequently led to the development of 3D models. Heinemann et al., 2013 established a three-dimensional co-culture model of osteoblasts and osteoclasts on a gel composed of silica, collagen and calcium phosphate (Heinemann et al., 2013); Tortelli et al. implanted mouse osteoblasts and osteoclasts precursors on porous ceramic scaffolds to establish a 3D bone model *in vitro* (Tortelli et al., 2009). Three-dimensional co-culture models simulate the arrangement and distribution of cells *in vivo* by culturing cells on a three-dimensional matrix, which is commonly used in hydrogels and solid scaffolds. The hydrogels and solid scaffolds have their own advantages and disadvantages. While the transparency of the soft hydrogel matrix is high and can be imaged by optical instruments, its mechanical strength is low and does not replicate the elastic properties of the natural bone environment. Studies have shown that the elastic modulus of the culture matrix provides the necessary conditions for guiding osteogenic differentiation (Reilly and Engler, 2010). Although solid-state scaffolds promote cell differentiation, the selection of solid-state 3D scaffolds as the substrate also restrict the use of optical analysis methods, owing to their limited optical penetration. The selected matrix materials should be as close as possible in composition to natural bone, in order to provide a suitable microenvironment for the cells. The methods that 3D printing techniques for bone remodeling research contain extrusion, laser-assisted, and inject bioprinting (Murphy and Atala, 2014; Magin et al., 2016; Wang et al., 2021). Three different matrix materials respectively are polymers (Thadavirul et al., 2014; Liu et al., 2021a; Nahanmoghadam et al., 2021) (polycaprolactone, polyether-ether-ketone, gelatin, and Alginate Hydrogel, etc.), Bioceramics (Seidenstuecker et al., 2019; Wei et al., 2022) (tricalcium phosphate, hydroxyapatite, and calcium sulfate, etc.), and Metals (Chou et al., 2013; Tian et al., 2022) (Iron, manganese, and molybdenum). Although other material has lots of benefits, the polymers (Bharadwaz and Jayasuriya, 2020; Fakhri et al., 2020; Eldeeb et al., 2022) that have biocompatibility, biodegradability, and interaction with cells, and bioactive ceramics (Ma et al., 2018; Zafar et al., 2019; Rahimnejad et al., 2021) that excellent mechanical properties and biocompatibility, being popular in 3D printed bone reconstruction studies. Therefore, combining polymers and bioactive ceramics to 3D print scaffolds for bone reconstruction are more routinization (Nyberg et al., 2017; Curti et al., 2020). By combining different materials and adjusting proportions, researchers explore models suitable for bone reconstruction studies. In this study, an extrusion bioprinting method was used to combine sodium alginate, gelatin, and hydroxyapatite into a composite biomaterial. Osteoblast precursor cells (MC3T3-E1) and osteoclast precursor cells (RAW264.7) were chosen as seed cells (Jingxuan et al., 2019; Xie et al., 2022; Yuan et al., 2022). A model for studying bone reconstruction mechanisms was constructed via 3D bioprinting. It provided a promising way for investigating complications such as in clinical settings, organs in subsequent studies.

## 2 Experimental method

### 2.1 Preparation of hydrogel scaffold

#### 2.1.1 Preparation and disinfection of3D bioprinting materials

SA (8%, w/v), Gel (5%, w/v), and HA (4%, w/v)were dissolved in aseptic phosphate buffered saline (PBS), and the printing material was prepared in sol state. The materials were put into an airtight container and sterilized at a temperature of 70°C for 30 min. The sterilization was repeated 3 times, at a frequency of once every 24 h. After sterilization, the 3D bioprinting matrix material was placed at 4°C for further use. For the printing of acellular scaffolds, the mixed sol of sodium alginate, gelatin and hydroxyapatite was printed and extruded continuously in a flat plate by a 3D biological printer (BioScaffolder 2.1, Germany) under the control of computer aided design, driven by air pressure. The crosslinking liquid CaCl_2_ was added, and the extruded sol scaffold instantly crosslinked and solidified in the cross-linking liquid CaCl_2_ solution, which completed the transformation from sol to gel under aseptic conditions. The printing parameters used were: print needle diameter 0.41 mm, extrusion pressure 120 kPa, bracket side length 10 mm, layer height 0.16 mm each bracket 4 layers, 7 lines per layer, 90° angle printing, while the barrel is heated at 37°C.

### 2.2 Biocompatibility of scaffold materials

#### 2.2.1 Preparation of leaching solution of scaffold material at different time points

The scaffold was placed in a centrifuge tube, followed by the addition of 2 mL DMEM/F12 medium containing 10% FBS. They were incubated at 37°C with shaking at 100 rpm/min on a rotatory shaker incubator. The scaffold extracts were collected on days 1, 2, 4 and 8. Fresh medium (2 mL) was added to the centrifuge tube at each instance of sample collection. For the cell proliferation toxicity test, the cell suspension (MC3T3-E1 cells; RAW264.7 cells) with 1 × 10^5^ cells per milliliter was added to a 96-well plate (100 μL/well) and cultured overnight in a cell incubator at 37°C under 5% CO_2_. The culture medium was removed and the scaffold extracts (200 μL/well) obtained at different sampling time points were added, with 3 wells in each group. Only DMEM/F12 culture medium was added in the negative control group. After 48 h of culture, the media in the wells was replaced with fresh medium (200 μL/well). This was followed by the addition of 20 μL CCK-8 solution to each well. The cells were incubated in the cell incubator for 1–4 h and their absorbance was determined by an enzyme labeling instrument (BioTek synergy 2, America) at 450 nm.

#### 2.2.2 Cell proliferative activity (Calcein-AM/PI staining)

The hydrogel containing seed cells was printed in three dimensions, and the printed scaffolds were cultured for 48 h at 37°C in a cell incubator supplemented with 5% CO_2_. The Calcein and PI solutions were diluted to final concentrations of 5 μM and 1.5 μM, respectively, with 1× PBS buffer. The two diluents were mixed at a ratio of 1:1, before being added into the well containing the cell scaffold. After an incubation period of 30 min, the scaffold was washed twice with 1× PBS buffer. The cell growth on the scaffold was observed using a fluorescence microscope (Ts2R-FL Nikon, Japan), where the living cells stained green while the dead cells stained red.

### 2.3 Scaffold characterization and detection

#### 2.3.1 Scanning electron microscopic analysis of the scaffold

RAW264.7 cells were dropped on acellular scaffolds and cultured at 37°C for 48 h under 5% CO_2_ in an incubator. The surface morphology of the scaffolds was observed using scanning electron microscope. In addition, the 3D bioprinting matrix material containing seed cells was printed in three dimensions, and the printed scaffolds were cultured in an incubator at 37°C for 48 h under an atmosphere of 5% CO2. The cell adhesion on the scaffolds was observed using scanning electron microscope (Hitachi HT7700 Exalens, Japan).

#### 2.3.2 Measurement of scaffold porosity

The scaffold was placed into a measuring cylinder containing 20 mL anhydrous ethanol. Before the scaffold was placed, the initial volume of absolute ethanol was recorded as V1. After the scaffold was allowed to soak for 1 day, the volume of absolute ethanol was recorded as V2. After the scaffold was removed, the volume of absolute ethanol was recorded as V3. The porosity of the scaffold was measured and the average value was taken. The formula used for calculating the scaffold porosity was: P = (V1-V3)/(V2-V3) × 100%.

#### 2.3.3 Determination of the scaffold degradation rate

The initial dry weight W0 of each scaffold was recorded. Subsequently, the scaffold was placed in a 1.5 mL centrifuge tube, followed by the addition of 1 mL of normal saline or simulated body fluid (SBF). The centrifuge tube was placed in a 37°C incubator, and the saline or SBF was replaced every week. On days 7, 14, 21 and 28, the scaffold was carefully transferred to a 24-well plate and washed 5 times with deionized water. The bracket was transferred to sterile cotton yarn for 5 min in order to allow the yarn to absorb the excess deionized water, before being freeze-dried for 48 h. After the scaffold was completely dry, the dry weight W1 at each time point was recorded, and the relative weight loss rate *in vitro* was calculated: P = (W0-W1)/W0 (n = 4).

#### 2.3.4 Determination of the scaffold swelling rate

The scaffold were freeze-dried and weighed (W0). Next, the scaffolds were soaked in normal saline/the cell culture media and kept at 37°C. The scaffolds were removed every 30 min and their surface was dried with filter paper. The expansion weight (WX) of each scaffold was recorded continuously for 3 h. The water absorption expansion rate was calculated according to the following formula: water absorption expansion rate (%) = (mass of the scaffold after water absorption WX-scaffold dry mass W0)/support dry mass W0 × 100%.

#### 2.3.5 Scaffold compression testing

The compression strength and modulus of the scaffold were tested using an electronic universal testing machine (UTM2102, Shenzhen Sun Technology Co., Ltd.). The scaffold, with dimensions of 10 mm × 10 mm × 3 mm, underwent compression at a rate of 5 mm per minute under displacement control. Stress was calculated as the applied force divided by the contact surface area, and strain was calculated as the displacement divided by the total height of the scaffold. After processing, stress-strain curves were obtained. The modulus and strength compression were assessed from the initial linear region and end of stress–strain curves, respectively.

### 2.4 Cell culture

#### 2.4.1 Culture of RAW267.4 cells and MC3T3-E1 cells

RAW264.7 cells and MC3T3-E1 cells were obtained from the Cell Bank of Chinese Academy of Sciences (Shanghai, China). RAW264.7 cells were cultured in DMEM medium supplemented with 10% fetal bovine serum and 1% penicillin-streptomycin, while MC3T3-E1 cells were cultured in α-MEM medium supplemented with 10% fetal bovine serum and 1% penicillin-streptomycin.

### 2.5 Verification of the feasibility of single culture, direct-contact co-culture and indirect-contact co-culture between scaffolds and cells

#### 2.5.1 Establishment of models of single culture, direct-contact co-culture and indirect-contact co-culture between scaffolds and cells

Three kinds of cellular hydrogels were configured (MC3T3-E1 cells + hydrogels; RAW264.7 cells + hydrogels; MC3T3-E1 cells + RAW264.7 cells + hydrogels). The material was printed using 3D bioprinting technology, with the aid of computer-aided design and driven by air pressure. The material was solidified immediately after printing by the addition of 5% CaCl_2_ solution. In addition, the scaffold model of indirect-contact co-culture was made by 3D bioprinting. Firstly, the hydrogel containing RAW264.7 cells was printed into two layers of scaffolds using 3D bioprinting technology. Next, the polycarbonate membrane with a pore diameter of 0.4 μm was affixed to the scaffolds, and the hydrogel containing MC3T3-E1 cells was quickly printed on the scaffolds using three-dimensional printing technology. During the printing process, 5% CaCl_2_ was slowly added to the bracket under the film in order to prevent the lower scaffold from collapsing. Immediately after the completion of three-dimensional printing, 5% CaCl_2_ was added to the scaffold above the polycarbonate film, in order to solidify the entire scaffold. All scaffolds were washed twice with preheated PBS. They were subsequently cultured in a 24-well plate containing culture medium in a cell incubator (37°C, 5% CO2) under aseptic conditions. The printing parameters used were as follows: the diameter of the print needle is 0.41 μm, the extrusion pressure is 120 kPa, the side length of the bracket is 10 mm, the height of each layer is 0.1 mm, 7 lines per layer, the number of printing layers is 4, the printing angle is 90°, and the printing syringe is kept at 37°C. It should be noted that in the 3D bioprinting model co-culture system, the pore size of polycarbonate membrane is 0.4 μm. Since it is less than 3.0 μm, cells do not migrate through the membrane; only compounds secreted or metabolized by cells pass through the membrane.

#### 2.5.2 Detection of related indexes of scaffold and cell culture alone, direct contact co-culture and indirect contact co-culture model

The scaffold model (2.5.1) was cultured for 24 h. The culture medium was replaced with fresh medium, and cytokines OPG (40 ng/mL) or RANKL (100 ng/mL) were added for a period of 6 days for induction. The expression of osteoblast and osteoclast markers (ALP/TRAP) was detected by qRT-PCR and ALP/TRAP enzyme activity detection kit, with the scaffold model without cytokines acting as the control group. The feasibility of the scaffold model was evaluated by determining whether the cells differentiated to the expected extent under the intervention of cytokines.

### 2.6 Statistics and analysis

All the experiments were performed in triplicates or quadruplicates. The data were expressed as mean and standard deviation. SPSS statistical software (IBM SPSS, Armonk, NY, United States) was used for inter-group *t*-test and analysis of variance. *p* < 0.05 was considered to be statistically significant.

## 3 Results

### 3.1 Preparation and characterization of hydrogel scaffolds

**FIGURE 1:**
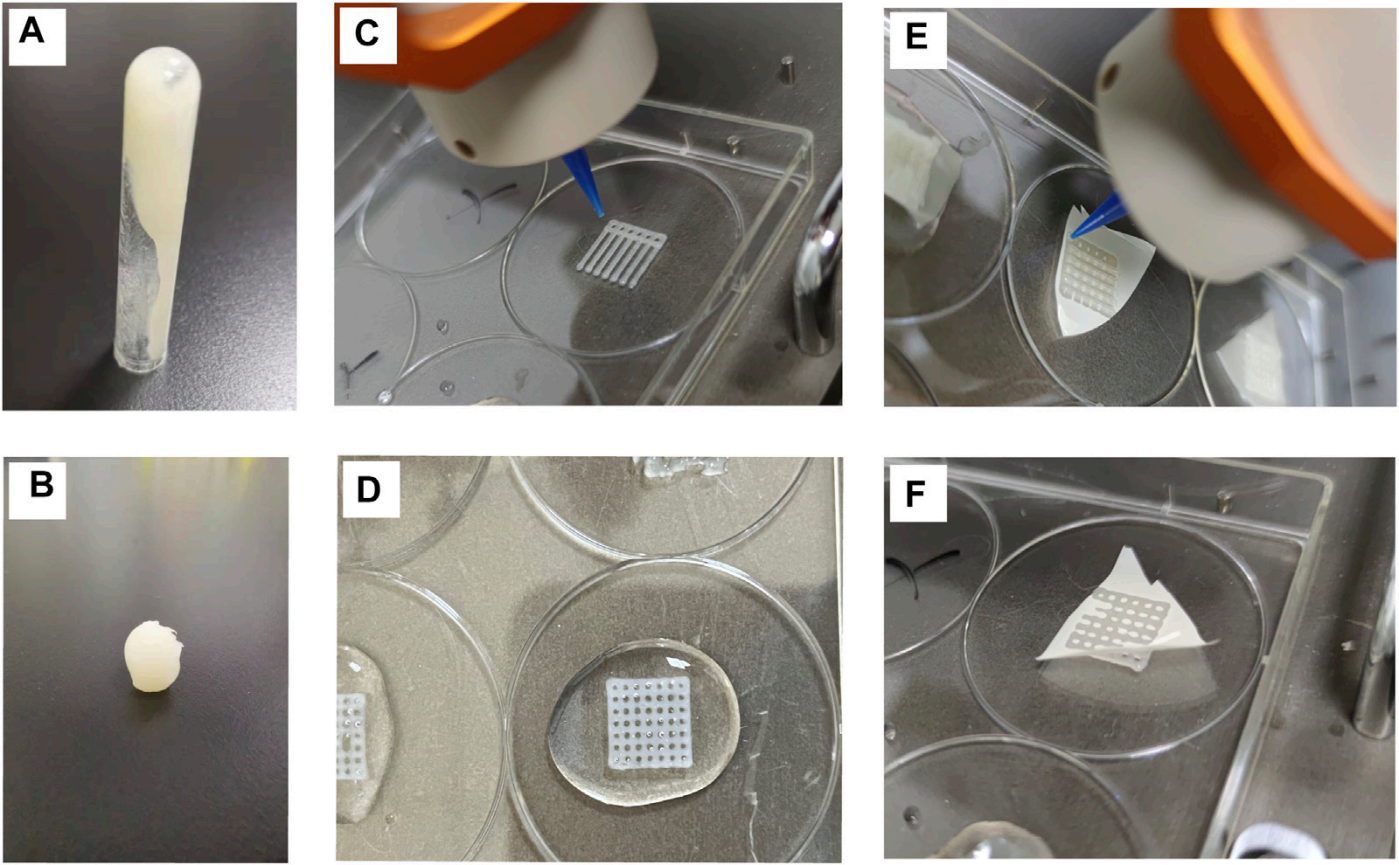
Characterization of hydrogel scaffold materials **(A)** Hydrogel materials with fluidity **(B)**. Crosslinked and cured hydrogel materials with CaCl_2_ **(C)**. 3D scaffold printing process **(D)**. Scaffolds cured by cross-linking with CaCl_2_ **(E,F)**. The printing process of covering polycarbonate film on a scaffold.

**FIGURE 2:**
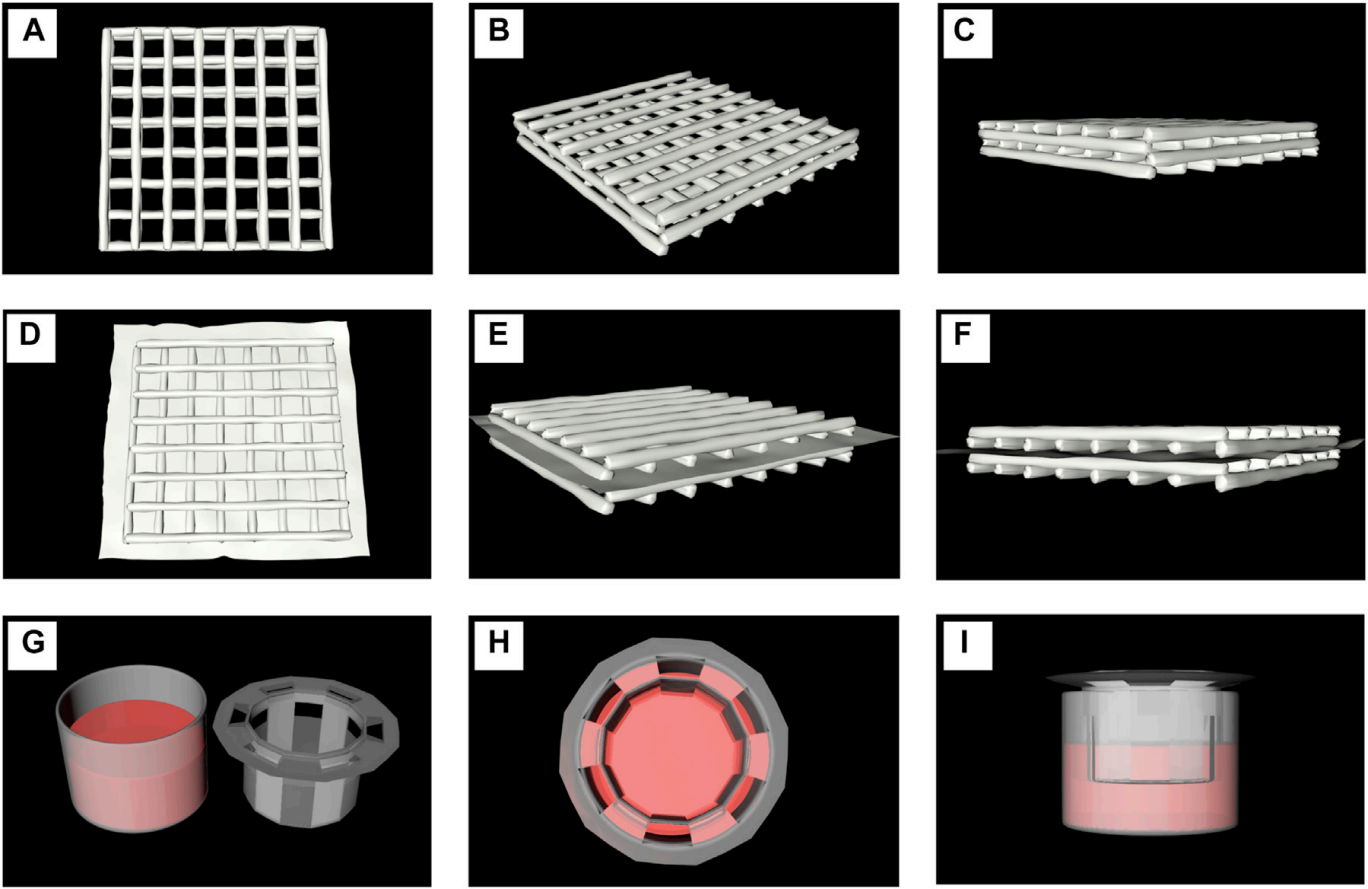
Model diagrams of different cell culture modes. **(A–C)**. The printed scaffolds co-cultutred with one of the cell line or the mixture of two types **(D–F)**. A model diagram using a polycarbonate membrane to separate two scaffolds printed by hydrogels containing different cells. **(G–I)** The indirect contact models of two kinds of cells at 2D level were tested in Transwell Petri dish.

The preparation method of the hydrogel for three-dimensional printing has been previously described. The hydrogel had a certain level of fluidity without the addition of CaCl_2_ (Figure 1A), thereby indicating that it had not been cured. The hydrogel material treated with CaCl_2_ could be molded into a specific shape (Figure 1B), thus indicating that the hydrogel material had solidified. Figure 1C shows the process of scaffold printing. After the printing was over, CaCl_2_ was quickly added to the scaffold (Figure 1D) in order to solidify it and prevent its collapse. The three-dimensional horizontal indirect contact culture model of the two types of cells is shown in Figures 1E,F. After two layers of scaffolds were printed, they were covered with polycarbonate film (Figure 1F). The other two layers of scaffolds were printed on the polycarbonate membrane (Figure 1E). The models of different cell culture modes are shown in Figure 2.

### 3.2 Biocompatibility of three-dimensional printed scaffolds

**FIGURE 3:**
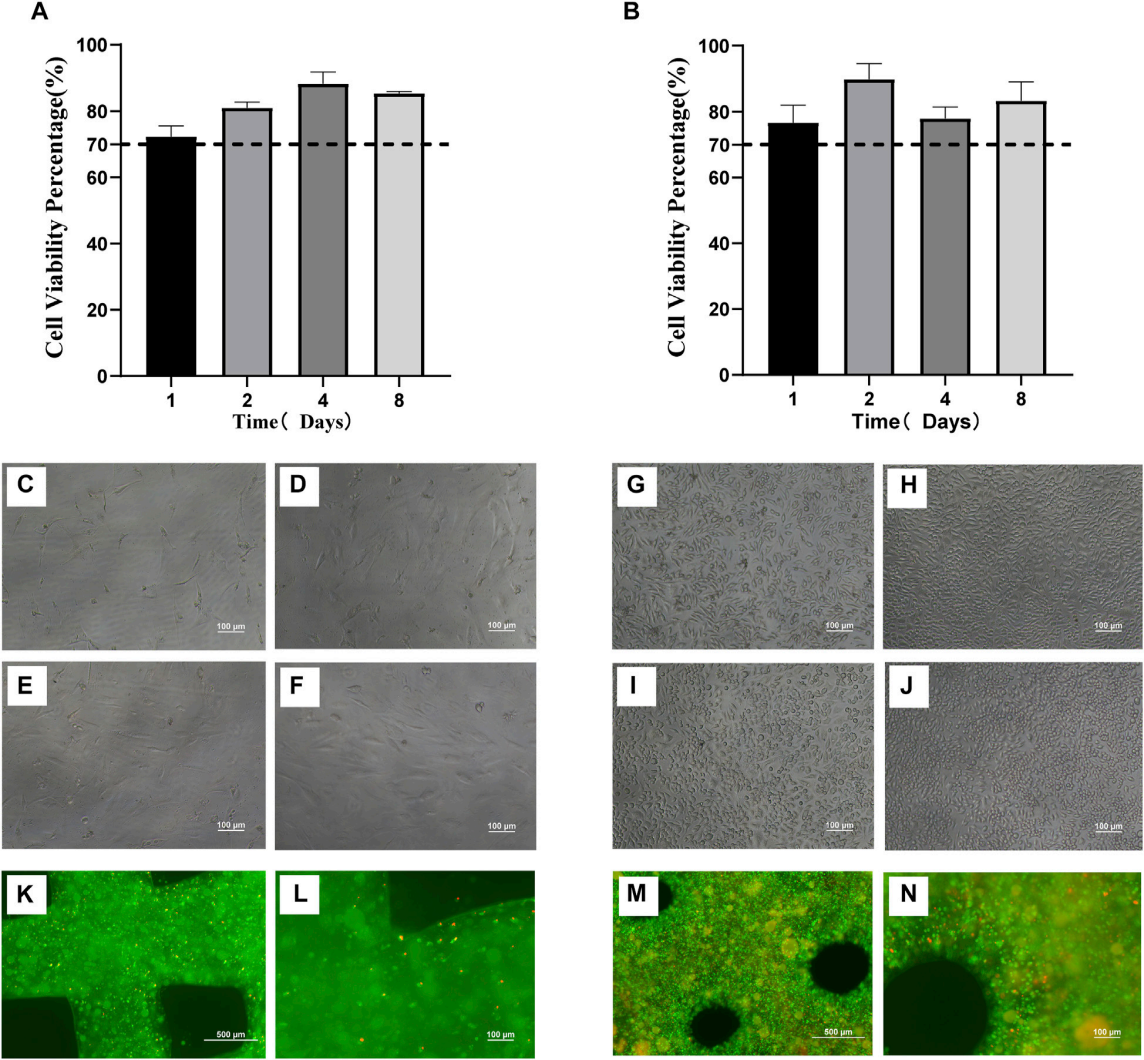
Biocompatibility of three-dimensional printed scaffolds **(A)**. Proliferative activity of MC3T3-E1 cells cultured in scaffold immersion solution **(B)**. Proliferative activity of RAW264.7 cells cultured in scaffold immersion solution. **(C–F)** The growth status of MC3T3-E1 cells in the scaffold extract collected on days 1, 2, 4 and 8 (100×). **(G–J)**. The growth status of RAW264.7 cells in the scaffold extract collected on days 1, 2, 4 and 8. (100×). **(K,L)** The scaffolds containing MC3T3-E1 cells cultured for 48 h were stained with Calcein-AM/PI under 40x microscope and 100x microscope. The living cells were stained green while the dead cells were stained red. **(M,N)** The scaffolds containing RAW264.7 cells cultured for 48 h were stained with Calcein-AM/PI and observed under 40x microscope and 100x microscope. The living cells were stained green while the dead cells were stained red.

In order to detect the toxic effects of scaffolds on cells, the proliferative activity of cells cultured in scaffold immersion solution (2.2.1) was detected by the CCK-8 method, while the activity of cells in scaffolds was detected by live/dead staining. The *in vitro* cytotoxicity test showed that the scaffold extract slightly decreased the cell survival rate. The proliferative activity of MC3T3-E1 cells (Figure 3A) and RAW264.7 cells on the day 1 (Figure 3B) after culture with scaffold extract was lower than that of other groups, but also more than 70%. Moreover, a proliferative activity of more than 90% was observed in MC3T3-E1 cells collected on day 4 (Figure 3A) and RAW264.7 collected on day 2 (Figure 3B) of culture with scaffold extract. Figures 3C–J show the growth of MC3T3-E1 and RAW264.7 cells in the scaffold immersion solution collected on days 1, 2, 4 and 8. The growth of cells as well as their morphology was found to be normal. Calcein-AM/PI reagent was used to stain the scaffolds co-cultured with cells for 48 h, with the living cells staining green and dead cells staining red (Figure 3K-N). The growth of MC3T3-E1 cells in the scaffold is shown in Figure 3K-L. Most of MC3T3-E1 cells were found to be alive, although the cell density was low owing to the small amount of inoculation, long cell passage cycle and short growth time. Most of the RAW264.7 cells were also alive (Figure 3M-N), and the cell density was relatively high owing to the higher amount of inoculation and relatively short cell passage cycle, when compared to the MC3T3-E1 cells.

### 3.3 Characterization of scaffolds

**FIGURE 4:**
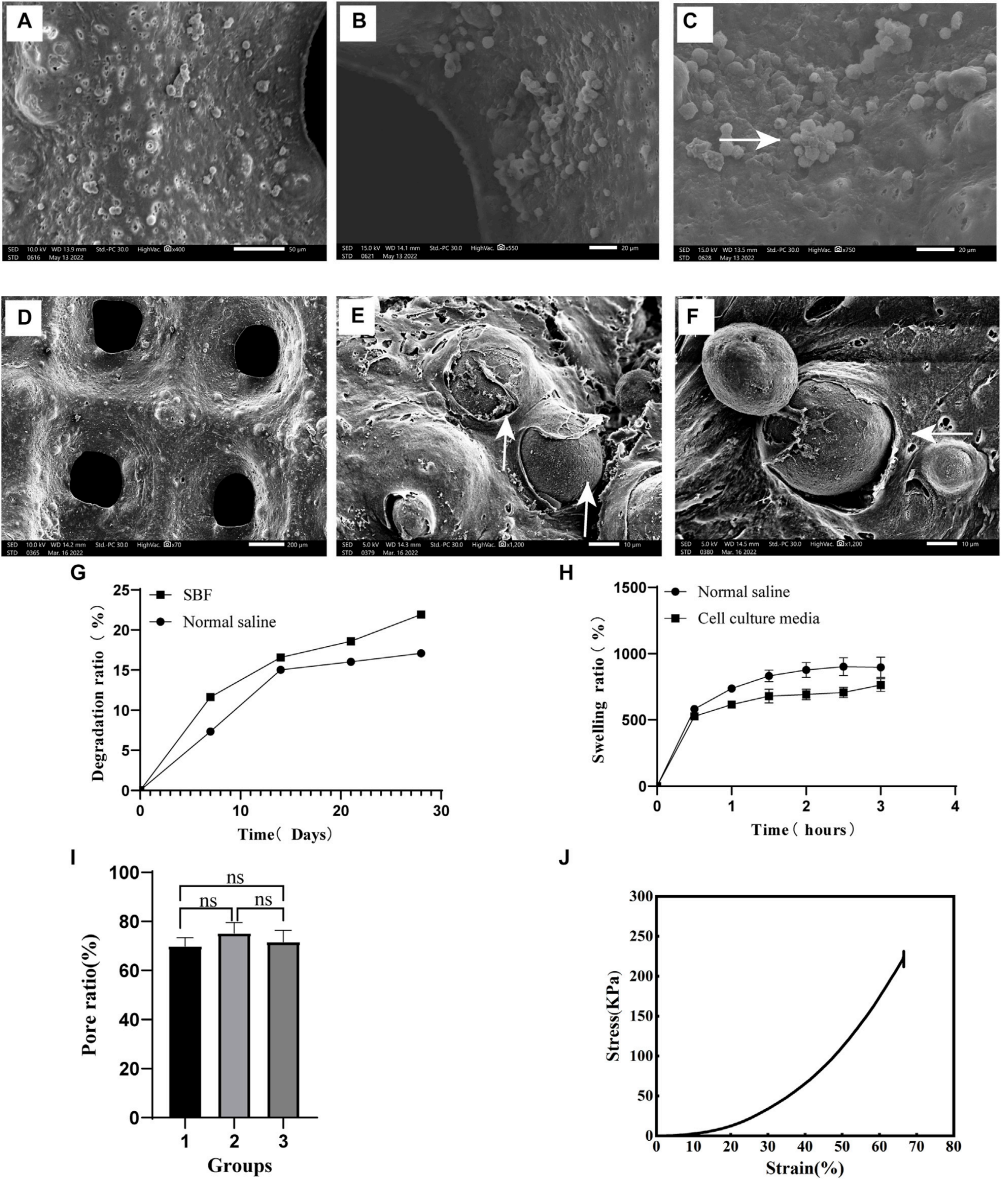
Scaffold characterization and detection **(A)**. The surface of the scaffold (400×) **(B)**. Pore of scaffold (500×) **(C)**. After the cell suspension was added to the scaffold, the cells adhered to its surface and grew in clusters (750×), as shown by the white arrow. **(D)** The surface morphology of the scaffolds co-printed with cells (70×) E and **(F)**. After 48 h of culture with the scaffold co-printed with cells, the cells adhered closely to the scaffold, as shown by the white arrow at 1500× **(E)** and 1200× **(F)** magnifications. **(G)** The degradation rate of scaffolds in different liquids. **(H)** Swelling ratio of scaffold immersed for different time periods. **(I)** Porosity of the scaffold. **(J)** Stress-Strain curve.

The surface of the scaffold was found to be rough and porous during analysis by scanning electron microscopy (SEM). When RAW264.7 suspension was dripped on the surface of the scaffold, the cells adhered to it (Figures 4A–C). The co-printed electron microscopic images of RAW264.7 cells and scaffolds show that the cells are closely attached to the scaffolds (Figures 4D–F). The scaffold degradation ratio was fast in the first week (7.33% ± 0.06%) and the second week (15.03% ± 0.15%) in saline, before gradually leveling off. In the simulated body fluid (SBF), the degradation ratio was faster in the first week (11.63% ± 0.4%) than in saline, before gradually leveling off (Figure 4G). The swelling rate of the scaffold in normal saline in the first hour rapidly increased to 736.33% ± 21.54%. In the next 2 h, it slowed down and gradually reached the equilibrium of water absorption at 876.92% ± 60.56% (Figure 4H). Similarly, the swelling rate of the scaffold in the cell culture media reached 616.12% ± 29.59% at the first hour. During the next 2 h, its swelling rate gradually slowed down and gradually reached the equilibrium of absorption (723.08% ± 51.8%) (Figure 4H). The porosity of the scaffolds showed no significant difference among the three groups, with a consistent porosity of 72.21% ± 4.38%. The porosity aided the transport and exchange of nutrients, cytokines and cell metabolic wastes, which met the essential design criteria of tissue engineering scaffolds (Figure 4I). The stress-strain curve of the scaffold is depicted in Figure 4J. The compressive modulus measures 615.27 ± 33.47KPa, while the compressive strength is recorded at 229.08 ± 2.32 KPa.

### 3.4 Feasibility verification of single culture model of scaffold cells

**FIGURE 5:**
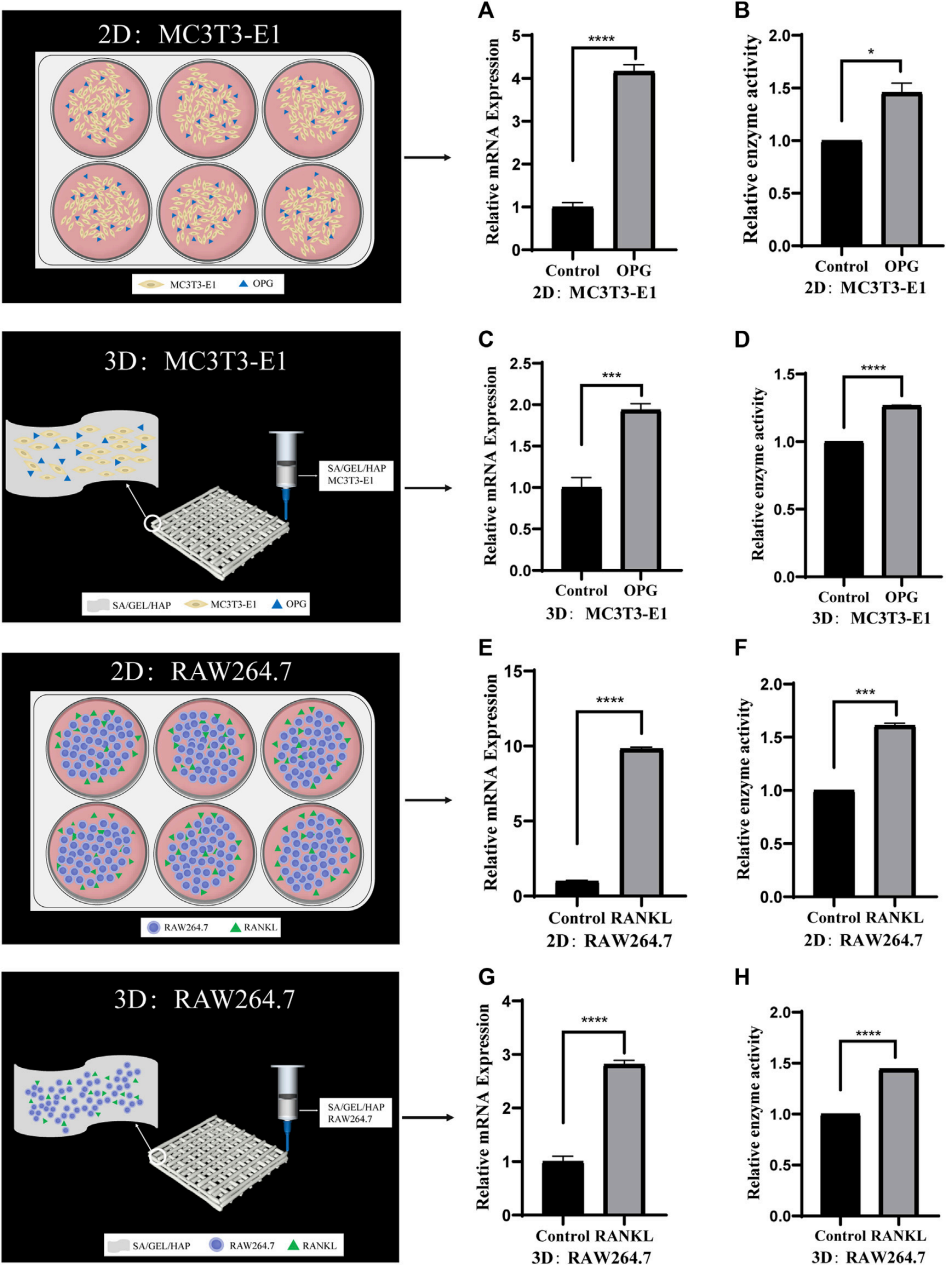
Comparison of the activities of specific genes and enzymes induced by cytokines in cells cultured separately under 2D and 3D conditions. **(A,B)** The relative ALP gene expression and enzyme activity of MC3T3-E1 cells induced by OPG in 2D culture. **(C,D)** The relative ALP gene expression and enzyme activity of MC3T3-E1 cells induced by OPG in 3D culture. **(E,F)** The relative TRAP gene expression and enzyme activity of RAW264.7 cells induced by RANKLin 2D culture. **(G,H)** The relative TRAP gene expression and enzyme activity of RAW264.7 cells induced by RANKL in 3D culture. (*p < 0.05, ***p < 0.001, ****p < 0.0001).

In order to verify the feasibility of individual culture of cells in the scaffold, we induced the co-culture of cells at 2D and 3D levels. MC3T3-E1 cells were induced with cytokine OPG for 6 days, and the relative gene expression and enzyme activity of ALP were detected. RAW264.7 was induced by RANKL for 6 days, and the relative gene expression and enzyme activity of TRAP were detected (Figures 5A–H).

### 3.5 Feasibility of direct contact between MC3T3-E1 and RAW264.7 cells and co-culture with scaffolds

**FIGURE 6:**
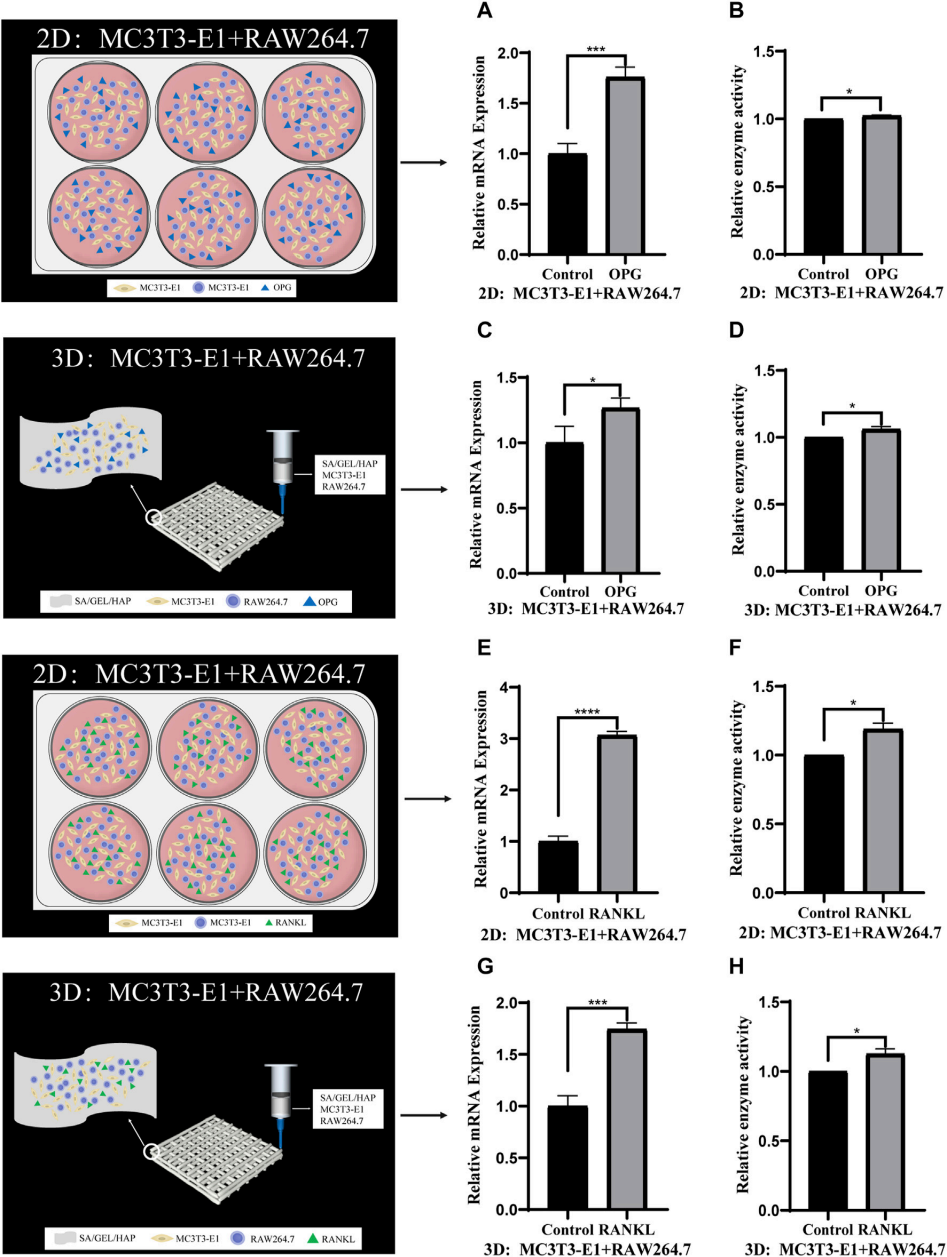
Comparison of gene expression and enzyme activities induced by cytokines in direct contact culture of two kinds of cells under 2D and 3D conditions. **(A,B)** The relative gene expression and enzyme activity of ALP in MC3T3-E1 cells and RAW264.7 cells induced by OPG in 2D culture. **(C,D)** The relative ALP gene expression and enzyme activity of MC3T3-E1 cells and RAW264.7 cells induced by OPG in 3D culture. **(E,F)** The relative TRAP gene expression and enzyme activity of MC3T3-E1 cells and RAW264.7 cells induced by RANKL in 2D culture. **(G,H)** The relative TRAP gene expression and enzyme activity of MC3T3-E1 cells and RAW264.7 cells induced by RANKL in 3D culture. (*p < 0.05, ***p < 0.001, ****p < 0.0001).

To verify the feasibility of direct contact between MC3T3-E1 and RAW264.7 cells and co-culture with scaffolds, we also induced the scaffolds of the two types of cells in direct contact at 2D and 3D levels. Cell scaffolds were induced by cytokine OPG (40 ng/mL)/RANKL (100 ng/mL) for 6 days, and the relative gene expressions and enzyme activities of ALP/TRAP were detected (Figures 6A–H) .

### 3.6 Verification of the feasibility of indirect contact with MC3T3-E1 and RAW264.7 and co-culture with scaffolds

**FIGURE 7:**
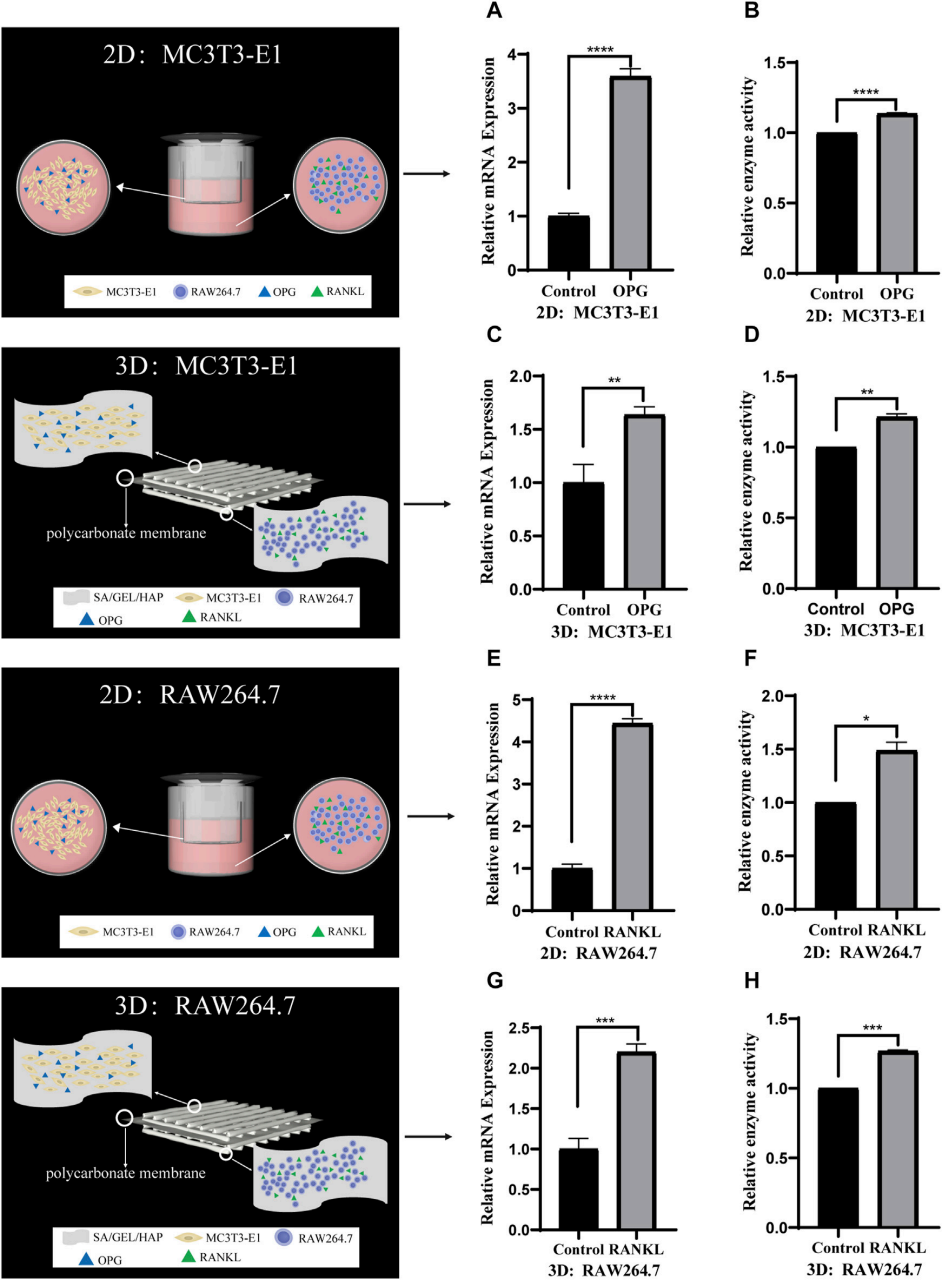
Comparison of the gene expression and enzyme activities induced by cytokines in indirect contact culture of two kinds of cells under 2D and 3D conditions. **(A,B)** The relative ALP gene expression and enzyme activity of MC3T3-E1 cells induced by OPG in 2D culture. **(C,D)** The relative ALP gene expression and enzyme activity of MC3T3-E1 cells induced by OPG in 3D culture. **(E,F)** The relative TRAP gene expression and enzyme activity of RAW264.7 cells induced by RANKL in 2D culture. **(G,H)** The relative TRAP gene expression and enzyme activity of RAW264.7 cells induced by RANKL in 3D culture. (*p < 0.05, **p < 0.005, ***p < 0.001, ****p < 0.0001).

To verify the feasibility of indirect contact with MC3T3-E1 and RAW264.7 cells and co-culture with scaffolds, we induced cells at 2D and 3D levels. MC3T3-E1 cells in indirect contact with RAW264.7 cells were induced with cytokine OPG for 6 days, and the relative gene expression and enzyme activity of ALP were detected. RAW264.7 cells in indirect contact with MC3T3-E1 cells were induced with cytokine RANKL for 6 days, and the relative gene expression and enzyme activity of TRAP were detected (Figures 7A–H).

## 4 Discussion

The occurrence of bone resorption is attributed to changes in the bone remodeling balance. Human and animal bone tissues are constantly reconstructed, and the process of bone reconstruction includes bone decomposition and absorption and new bone formation. Osteoclasts are responsible for bone decomposition and resorption, while osteoblasts are responsible for new bone formation. Osteoclasts adhere to the surface of old bone. They secrete acids and proteases to dissolve minerals and digest bone matrix, respectively, and form bone resorption lacunae. Subsequently, osteoblasts migrate to the absorbed site, where they secrete and mineralize the bone matrix in order to form new bone. Therefore, the balance between osteoclastogenesis and osteogenesis is the key to maintaining normal bone mass. The RANKL-RANK-OPG axis, which is composed of the receptor activator of NF- κ B Ligand (RANKL), receptor activator of NF- κ B receptor activating factor (RANK) and osteoprotegerin (OPG), is a key factor in the regulation of bone resorption and bone formation (Infante et al., 2019). The main activating factors in osteoclast differentiation are the monocyte colony stimulating factors M-CSF and RANKL, while the inhibitory factor is OPG. RANK is the only receptor of RANKL and is a key to its proper functioning. OPG competes with RANK to bind RANKL, inhibit osteoclast formation, reduce bone resorption and promote bone formation (Boyce and Xing, 2008; Anesi et al., 2019; Udagawa et al., 2021; Velletri et al., 2021). Although many studies have been reported on the RANKL-RANK-OPG signaling system, because the systemic effects of drugs and other interventions vary from person to person. Hence, results for the same intervention process may not be reproducible, thereby affecting the study of the mechanism of bone resorption.

The biological ink used for 3D bio-printing should have the necessary characteristics including non-toxicity, printability, biocompatibility and controllable biodegradability. Hydrogels hold considerable promise as bio-inks for use in 3D biological printing. Hydrogels provide a stable biomimetic microenvironment for cell adhesion, migration, proliferation and differentiation; hence, they are widely used in 3D bioprinting as cell-loaded materials. The three-dimensional culture microenvironment of the scaffold can simulate the natural extracellular matrix, wrap the cells and promote the expression of bone markers. The ideal biological scaffolds should be non-toxic and biocompatible. It should have specific characteristics including controllable biodegradability, low immunogenicity, biomechanical strength, optimum porosity and pore size, and the ability to induce cell or tissue differentiation (Zheng et al., 2019; Mirkhalaf et al., 2023). The hydrogels prepared using sodium alginate, hydroxyapatite and gelatin have the potential to be used for bone defect repair. Sodium alginate and gelatin are natural organic polymers with acceptable levels of temperature sensitivity, water uptake capacity and cytocompatibility. Sodium alginate is often used as a drug carrier in biological tissue engineering (Chang et al., 2021; Lee et al., 2021). Gelatin is the product of hydrolysis of collagen. It can be dissolved and gelated in water and has excellent mechanical properties and biocompatibility (Purohit et al., 2020). Gelatin also promotes cell growth and differentiation and improves tissue regeneration ability, thereby promoting bone formation (Zheng et al., 2019; Purohit et al., 2020). Hydroxyapatite is a kind of bioactive ceramic, with the chemical composition and structure being similar to that of natural bone tissue (Frezzo and Montclare, 2016; Koupaei and Karkhaneh, 2016; Abdul Halim et al., 2021; Udagawa et al., 2021; Ielo et al., 2022). Some studies have shown the formation of calcified nodules of different sizes on the surface of the scaffold after soaking hydroxyapatite with fetal bovine serum (FBS) for a period of time (Kokubo and Takadama, 2006). Hydroxyapatite may be loaded with drugs in order to regulate the proliferation, migration and differentiation of osteoblasts. It is an important inorganic material for bone repair with excellent osteogenic potential (Biedrzycka et al., 2021; Costa-Pinto et al., 2021; Hussin et al., 2021).

The composite biological ink used in this study contains a specific proportion of sodium alginate, gelatin and hydroxyapatite for 3D biological printing. The configured printing material has fluidity without CaCl_2_, which enables the printing material to adjust the viscosity and promote the stable formation of filaments. The printing material with CaCl_2_ can be molded into a specific shape, the scaffold will not collapse with pores after CaCl_2_ dropping. The primary consideration in the preparation of ideal biological scaffolds is its cytotoxicity and biocompatibility. *In vitro* cytotoxicity assays have confirmed that the scaffold extract leads to a slight reduction in the cell survival rate; however, the lowest proliferative activity was still more than 70%. Over time, the cell proliferative activity reached more than 90%. According to the International Organization for Standardization (ISO) 10,993–5 procedure, the extract of biomaterials is considered non-toxic when the cell survival rate is more than 70%. Studies on the cell state, survival rate and proliferation activity of MC3T3-E1 and RAW264.7 cells grown in scaffold immersion solution for different time periods showed that sodium alginate/gelatin/hydroxyapatite scaffold material had little cytotoxicity, good biocompatibility and promoted cell growth. It should be noted that the cells in the scaffold grow in a three-dimensional environment, hence the photos collected only represent the cells in a certain layer of the scaffold. Since the cells are at different levels, the fluorescence intensity of the observed cells is not uniform. The surface of the material has a great influence on the adhesion and proliferation of cells and the establishment of tissue or scaffold (Mandrycky et al., 2016). The multi-layer micropore structure on the surface of the material contributes to the biological behavior of cells (van Lenthe et al., 2007; Wang et al., 2023). The presence of pores promote cell migration, integration with host tissue, and angiogenesis (Vallet-Regí et al., 2012). Scanning electron microscopy studies have shown that the scaffold has a rough multi-layer microporous structure, which not only increases its surface area, but also facilitates the adhesion of cells to the scaffold. Studies exploring the stability of scaffolds under physiological conditions are very important for subsequent biological experiments. Biodegradable scaffolds can provide sufficient space for tissue and cell growth when implanted *in vivo* or during wound healing. Gelatin or sodium alginate, when used alone, can dissolve and lose their 3D structure within a few hours in PBS. However, when combined with materials like bioactive glass or polycaprolactone, they can form hydrogel scaffolds maintaining their 3D structure for at least 1 month (Rehman et al., 2019; Wu et al., 2019; Entekhabi et al., 2020; Ehterami et al., 2021). It has been discovered that the addition of gelatin and phenol significantly reduces the degradation rate of sodium alginate hydroge (Firouzi et al., 2020). Gelatin and polycaprolactone hydrogel scaffold exhibit a degradation rate of about 40% on the 30th day (Ehterami et al., 2021). Gelatin/Methacryloyl/Reduced graphene oxide hydrogel scaffold has a degradation rate of about 50% on the 28th day (Rehman et al., 2019). Therefore, in this study, the biodegradability of the scaffold in normal saline and simulated body fluids was tested. The results confirmed that the scaffold could maintain its three-dimensional structure under physiological conditions for at least 28 days, which meets the essential criteria of several medical and tissue engineering applications. Water absorption is an important characteristic of the scaffold, since a high water absorption capacity can function in a manner similar to biological tissues in medical applications, thereby leading to the absorption of exudates or toxic components. In line with previous reports, the swelling rate of the scaffold increased rapidly in the first hour after soaking in normal saline, before slowing down and gradually reaching the equilibrium of water absorption (Pan et al., 2019; Rehman et al., 2019). To sum up, the scaffold has good swelling ability, which provides a moist growth environment for cells and increases the exchange of nutrients and oxygen needed for cell survival. Similarly, the pore of the scaffold plays an important role in the transport and exchange of nutrients, cytokines and cellular metabolic wastes. A study of the scaffold porosity showed that the scaffold met the essential criteria of tissue engineering. While not as strong as ceramics, metals, and real bones in terms of compressive strength (Biggemann et al., 2018; Vedhanayagam et al., 2022), mechanical testing of the scaffold confirmed its significant compressive strength, consistent with previous research findings (Williams et al., 2005). Studies suggest that the compressive strength and modulus of the scaffold may decrease with an increase in hydroxyapatite content (Nahanmoghadam et al., 2021).

Although many studies have reported on the role of osteoblasts and osteoclasts in bone remodeling, the underlying mechanism has not been fully elucidated. Although some of the studies are based on a single cell model (Arora et al., 2020; Kamiya et al., 2020), it is obviously not enough to study the mechanism of bone remodeling. A stable co-culture system of osteoblasts and osteoclasts is vital for the study of the regulatory mechanism of bone remodeling. There are two types of cell co-culture—direct and indirect contact. Direct contact can be defined as the interaction between two different kinds of cells through synaptic contact. Takahashi et al., 1988 established a model of direct co-culture of primary mouse skull osteoblasts and mouse spleen cells, in order to study the effect of osteoblast contact on osteoclast formation (Takahashi et al., 1988). Zhao et al. used the method of direct co-culture of mouse osteoblast-like MLO-Y4 cells and bone marrow cells to study the effect of direct contact with osteoblasts on the formation and activation of osteoclasts (Zhao et al., 2002). In this study, In this study, mouse osteoblast precursor cells (MC3T3-E1 cells) and osteoclast precursor cells (RAW264.7 cells) were cultured at 2D and 3D (Figures 2A–F) levels, respectively, and induced by the addition of corresponding cytokines. The cell culture methods included single culture, direct contact co-culture, and indirect contact co-culture. The differentiation of MC3T3-E1 cells into osteoblasts, induced by OPG, was determined by detecting the expression of ALP in MC3T3-E1 cells. ALP is an important marker of osteogenic differentiation and bone mineral formation of biomaterials *in vitro* (Salarian et al., 2014; Vimalraj, 2020). The differentiation of osteoclasts induced by RANKL can be determined by detecting the expression of TRAP in RAW264.7 cells. TRAP is a specific index to reflect the activity of osteoclasts and bone resorption (Yao et al., 2011; Kamath et al., 2023).

When cultured alone, the ALP gene expression and enzyme activity of MC3T3-E1 cells cultured at 3D level were higher than those in the control group after OPG induction. This was consistent with the results of 2D level induction, which also confirmed the osteogenic ability of the scaffolds. Demirtas et al. reported that MC3T3-E1 cells co-cultured with composite hydrogel (chitosan/hydroxyapatite) and expressed osteogenic markers in the early and late. (Demirtaş et al., 2017). The expression of TRAP gene and its enzyme activity after the culture of RAW264.7 cells 3D level and induction by cytokine RANKL for 6 days was studied, with the results being consistent with those induced at 2D level. Similarly, studies have shown that Fmoc hydrogel modified with hydroxyapatite supports osteoclast formation of RAW264.7 cells *in vitro*, which has been confirmed by morphological changes and expression of osteoclast markers (Vitale et al., 2022). In this study, an analysis of the induction results of the two kinds of cells shows that the proliferation and differentiation induced by cytokine OPG/RANKL at 3D culture level is similar to that at the 2D culture level. Hence, the scaffold that we used could induce both osteogenesis as well as osteoclastogenesis, which indicated that the scaffold could induce new bone formation in a more efficient manner. At the 2D level, MC3T3-E1 cells and RAW264.7 cells were co-cultured in direct contact and induced by OPG/RANKL cytokines for 6 days. The results showed that the induced differentiation of the two kinds of cells was inhibited to different degrees after direct contact. This may be due to increased cellular communication, or the effect of cytokines on additional pathways, which needs to be investigated further. At the 3D level, the two types of cells were in direct contact and were subsequently cocultured with scaffolds for 6 days. The results were similar to those induced by direct contact at 2D level, although it was not as effective as the latter. At the same time, the induced differentiation was inhibited to different degrees when compared to the single culture at 3D level. On the contrary, some studies have shown that RAW264.7 cultured on scaffolds secrete additional factors to promote osteogenic differentiation in MC3T3-E1 cells (Liu et al., 2021b). One reason for this could be the difference in the scaffolds used, while the other could be that the mechanism of action of RAW264.7 secretions cultured on scaffolds on MC3T3-E1 cells is different from that of the two kinds of cells in direct contact. It can be concluded that the differentiation induced by direct contact co-culture at 3D level is similar to that at 2D level, but the induction effect is worse. Compared with the induced differentiation of cells cultured alone, the induced differentiation of cells was inhibited to varying degrees after direct contact with the two kinds of cells. Direct contact co-culture has certain shortcomings, including the inability to separate single cells from the co-culture system for follow-up biological experiments.

The limitations of direct contact co-culture can be overcome by indirect contact co-culture. Indirect contact is defined as two types of cells that are spaced apart but communicate indirectly through cytokine secretion. Bernhardt et al. developed an indirect co-culture model which provided a bone-like microenvironment for osteoblasts and osteoclasts precursors (Bernhardt et al., 2010); Li et al. developed a dynamic indirect co-culture model using Transwell chambers as spacers (Li et al., 2013). Similarly, in this study, we co-cultured MC3T3-E1 and RAW264.7 cells indirectly at the 2D level using the Transwell chamber culture method (Figures 2G–I). Indirect contact co-culture at 3D level was performed by the addition of a polycarbonate film in the middle of the scaffold, which separated the two kinds of cells involved in bone remodeling to either side of the matrix material (Figures 2D–F). This prevented migration between the two kinds of cells, although it still allowed the exchange of substances secreted or metabolized by the cells. The results of MC3T3-E1/RAW264.7 induction by cytokine OPG/RANKL at 2D level showed that, although the differentiation effect of indirect contact culture was not as good as that of single culture, it was higher than that of direct contact culture. The results at 3D level were similar to those at 2D level, with the differentiation effect being not as good as that at 2D level. Compared with direct contact culture at 3D level, the effect of induced differentiation improved to varying degrees and nearly reached the level of induced differentiation in single culture. The findings demonstrated that the interaction between cells inhibited their induced differentiation in direct contact co-culture.

## 5 Conclusion

In this study, it can be concluded that the hydrogel prepared from sodium alginate, gelatin and hydroxyapatite holds great promise as a 3D biological printing material owing to its biocompatibility, toxicity, surface morphology, porosity, degradation rate and water absorption expansion rate. Osteoblast precursor cell MC3T3-E1 and osteoclast premise cell RAW264.7 were induced in 2D, 3D and three different culture models. The results showed that cytokines could induce cell differentiation in 3D model, and that the construction method of 3D bone resorption mechanism model was feasible. However, there is still a reasonable difference between 3D bone resorption model and real bone matrix, which requires further investigation into the optimization of printing conditions and better material combinations. The 3D bioprinting research model of bone resorption mechanism has a great application prospect not only in basic research, but also in preclinical drug screening.

## Data Availability

The original contributions presented in the study are included in the article/Supplementary material, further inquiries can be directed to the corresponding author.

## Abbreviations

SA, Sodium alginate; HA, hydroxyapatite; Gel, gelatin; OPG, osteoprotegerin; RANKL, NF-κB ligand; ALP, alkaline phosphatase; TRAP, tartrate resistant acid phosphatase; SBF, simulated body fluid; PBS, phosphate buffered saline; SEM, scanning electron microscopy.
